# Bacterial Sepsis Pathogens and Resistance Patterns in a South Asian Tertiary Care Hospital

**DOI:** 10.7759/cureus.15082

**Published:** 2021-05-18

**Authors:** Zia U Rehman, Mohammad Hassan Shah, Muhammad Nauman Shah Afridi, Hafsa Sardar, Ahmad Shiraz

**Affiliations:** 1 Nephrology and Kidney Transplant Unit, Rehman Medical Institute, Peshawar, PAK; 2 Internal Medicine, Rehman Medical Institute, Peshawar, PAK; 3 Haematology, King’s College Hospital, London, GBR; 4 General Surgery, Ayub Teaching Hospital, Abbottabad, PAK; 5 Radiology, Hayatabad Medical Complex, Peshawar, PAK; 6 General Surgery, Hayatabad Medical Complex, Peshawar, PAK

**Keywords:** susceptibility, blood culture, resistant, sensitivity, histopathology & microbiology, antibiotic

## Abstract

Objective

The aim of this study was to determine common microorganisms causing septicemia and their antimicrobial sensitivities in patients admitted to a tertiary care hospital.

Methods

A cross-sectional study was conducted using clinical criteria to diagnose patients as having septicemia where blood for culture and sensitivity (CS) was sent to the laboratory of a tertiary care hospital, Rehman Medical Institute, (Peshawar), Pakistan, during 2019. All patients diagnosed with septicemia regardless of age and gender were included in the study. The blood CS report was collected after 7-14 days of inoculation. Data were recorded on structured performa and analyzed using SPSS Version 20 (IBM Corp.).

Results

A total of 176 patients, with a mean age of 2.92±1.32 years, fulfilled the criteria for sepsis with a mean age of 2.92±1.32 years. Among them, 61.9% were male and 38.1% were female. Among common bacterial isolates, *Staphylococcus aureus* was found in 37.5% of samples followed by skin contaminants (18.2%), methicillin-resistant *Staphylococcus aureus* (MRSA) (14.8%), and *Escherichia coli* in (11.4% cases). None of the antibiotics had susceptibility of more than 60%. Susceptibility to piperacillin/tazobactam and ampicillin/sulbactam was found in 21.5% and 14.6% of the samples, respectively, while in cephalosporins, cefoxitin’s susceptibility was 28.5%, whereas both ceftriaxone and cephazolin were equally effective in 19.4% cases. Furthermore, 38.9% of the samples were susceptible to ciprofloxacin and 24.3% to levofloxacin. The susceptibilities of amikacin and gentamicin in aminoglycosides were 56.3% and 47.2%, respectively, while that of imipenem and meropenem were 59.7% and 22.9%, respectively. Lastly, clindamycin had an efficacy in 42.4% of samples.

Conclusion

The susceptibility of bacterial isolates in septicemia to common antibiotics was low, thus risking therapeutic failure in septic patients. Widespread resistance may be due to the excessive use along with over-the-counter availability of antibiotics, which therefore requires regulation as it is an alarming situation.

## Introduction

Sepsis is a common problem associated with substantial mortality and significant consumption of healthcare resources [1]. It is the inflammatory response of the body toward the infectious agent, which, in turn, may cause severe sepsis and septic shock, which are its more severe forms [2]. Globally, the incidence of sepsis was 48·9 million in 2017, out of which 33.1 million had an underlying infectious disease and 15.8 million had other non-communicable diseases [3]. A greater part of the global sepsis burden is contributed by low- and middle-income countries, where 90% of sepsis deaths occur from pneumonia, meningitis, or other infections. The highest number of newborn and infant deaths occurring from sepsis is present in Asia and sub-Saharan Africa [4]. It has been reported that sepsis occurs in approximately 2% of all hospitalizations in developed countries; in intensive care units (ICUs), the prevalence ranged from 6% to 30% [5]. In Pakistan, the prevalence of septicemia due to bacteria was 14.75% [6]. An observational study in tertiary care hospitals reported that 40.7% of patients in tertiary care hospital ICUs had severe sepsis, while 59.3% developed septic shock [7]. The most common isolated bacteria among patients admitted in ICUs of tertiary care units included *Staphylococcus aureus* (36.38%) followed by* Escherichia coli* (18.28%) and methicillin-resistant *Staphylococcus aureus* (MRSA; 7.0%). Other bacteria included *Streptococcus faecalis*, *Salmonella typhi*, *Pseudomonas species*, and *Candida* species [6]. Overall mortality due to septic conditions is 12.5%, but it varies (ranging from 5.6% to 34.2%) by age and organ involvement [8]. Sepsis prevalence also varies by patient age. The majority of cases (58-65%) occur in older age groups. [9]. Martin et al. reported a 26% higher risk of death in elderly patients with sepsis during the first week of hospitalization compared to younger patients [10]. Similarly, sepsis is the leading cause of death globally in infants and patients aged over 50 years [11]. Sepsis incidence among children is strikingly high in early childhood [3]. A hospital-based study in Pakistan showed that 25% of admitted neonates had sepsis [12].

Mortality in sepsis is increased with ineffective antibacterial therapy, which, in turn, is linked to bacterial multidrug resistance. Preceding empiric antibiotic prescriptions, incomplete courses of drugs, and poor nutritional status increase the risk [13]. Given these influences, regional variation in microbiology and resistance patterns is expected. The present study aims to define organisms responsible for bacterial sepsis, as well as their antibiotic resistance patterns, in different age groups of patients admitted to Rehman Medical Institute (RMI), Peshawar, Pakistan.

## Materials and methods

This cross-sectional study identified patients ranging from neonates to elderly with a maximum age of 82 years over a period of one year from May 2019 to May 2020 who met sepsis criteria by the 2017 National Institute for Health and Clinical Excellence Sepsis guidelines at RMI [14]. Blood culture results including susceptibilities, patient demographics, and medical history were recorded. Sensitivity patterns were analyzed by age group, organism, and underlying diseases. SPSS Version 20 (IBM Corp., Armonk, NY) was used for data analysis.

## Results

**Figure 1:**
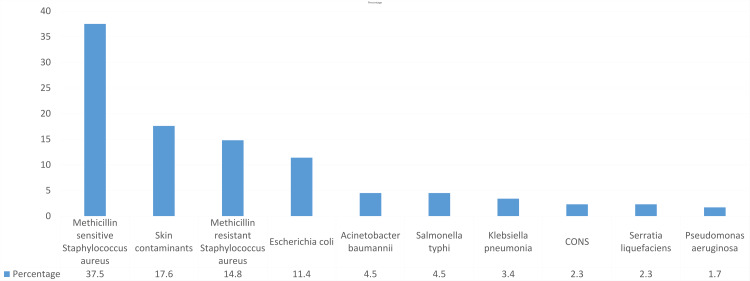
Common pathogens responsible for causing septicemia CONS, coagulase-negative Staphylococcal species

A total of 176 patients fulfilled the clinical criteria for sepsis, of whom 109 (61.9%) were male patients and 67 (38.1%) were female patients, with a mean age of 2.92±1.32 years. Among the isolated bacteria, *Staphylococcus aureus* was the most common (37.5%) followed by skin contaminants (17.6%) and MRSA (14.8%), whereas *Escherichia coli* accounted for 11.4% (Figure 1).

 

Of the 176 samples, 144 had clear results of sensitivity and resistance pattern, while 32 samples were deemed skin contaminants and excluded from further analysis, suggesting possible septicemia due to viral causes. In the penicillin group, only piperacillin/tazobactam and ampicillin/sulbactam had 21.5% and 14.6% susceptibilities, respectively. The susceptibility was very low for other penicillins. Among cephalosporins, cefoxitin was found effective in 28.5% of cases, while cephazolin and ceftriaxone both showed susceptibility in 19.4% of the cases. Among quinolones, 38.9% of the samples were susceptible to ciprofloxacin and 24.5% to levofloxacin, respectively. Among aminoglycoside, only amikacin and gentamicin were found effective in 56.3% and 47.2% of septicemia cases, respectively. Among carbapenems, imipenem and meropenem were found effective in 59.7% and 22.9% of the cases, respectively. Lastly, clindamycin showed susceptibility in 42.4% of samples.

In addition, susceptibility to vancomycin was 58.3% followed by chloramphenicol, which was found susceptible in 57.6% of the samples. Susceptibilities to other antibiotics included doxycycline (52.8%), minocycline (47.2%), co-trimoxazole (34.7%), and rifampicin (31.2%). On the other hand, very low susceptibilities were found for novobiocin, colistin, nalidixic acid, tigecycline, nitrofurantoin, and teicoplanin (Table 1).

**Table 1:** Sensitivity and resistance pattern (N=total number of cultures where antibiotic was tested)

	N	Resistant, N (%)	Susceptible, N (%)
Penicillin group			
Ampicillin	144	134 (93.1%)	10 (6.9%)
Amoxicillin/clavulanic acid	144	135 (93.75%)	9 (6.3%)
Amoxicillin	144	138 (95.8%)	6 (4.2%)
Ampicillin/sulbactam	144	123 (85.4%)	21 (14.6%)
Penicillin	144	142 (98.6%)	02 (1.3%)
Piperacillin/tazobactam	144	113 (78.5%)	31 (21.5%)
Cephalosporin group			
Cefaclor	144	127 (88.2%)	17 (11.8%)
Cefazolin	144	116 (80.6%)	28 (19.4%)
Cefoperazone or sulbactam	144	136 (94.4%)	8 (5.6%)
Cefoxitin	144	103 (71.5%)	41 (28.5%)
Cefotaxime	112	106 (94.6%)	6 (5.4%)
Ceftriaxone	143	115 (80.4%)	28 (19.6%)
Cefuroxime	136	111 (81.6%)	25 (18.4%)
Cefixime	143	142 (99.3%)	1 (0.7%)
Cefalexin	144	128 (88.9%)	16 (11.1%)
Cefepime	143	137 (95.8%)	6 (4.2%)
Fluoroquinolone group			
Ciprofloxacin	144	88 (61.1%)	56 (38.9%)
Moxifloxacin	144	130 (90.3%)	14 (9.7%)
Lomefloxacin	144	143 (99.3%)	1 (0.7%)
Levofloxacin	143	108 (75.5%)	35 (24.5%)
Aminoglycoside group			
Tobramycin	144	135 (93.8%)	9 (6.2%)
Fosfomycin	144	143 (99.3%)	1 (0.7%)
Gentamicin	144	76 (52.8%)	68 (47.2%)
Amikacin	144	63 (43.8%)	81 (56.2%)
Monobactam such as aztreonam	144	143 (99.3%)	1 (0.7%)
Carbapenem group			
Invanz ertapenem	144	139 (96.5%)	5 (3.5%)
Imipenem	144	58 (40.3%)	86 (59.7%)
Meropenem	144	111 (77.1%)	33 (22.9%)
Doripenem	144	133 (92.4%)	11 (7.6%)
Macrolide group			
Azithromycin	144	136 (94.4%)	8 (5.6%)
Clarithromycin	144	138 (95.8%)	6 (4.2%)
Clindamycin	144	83 (57.6%)	61 (42.4%)
Erythromycin	144	134 (93.1%)	10 (6.9%)
Other antibiotics			
Chloramphenicol	144	61 (42.4%)	83 (57.6%)
Co-trimoxazole	144	94 (65.3%)	50 (34.7%)
Doxycycline	144	68 (47.2%)	76 (52.8%)
Vancomycin	144	60 (41.7%)	84 (58.3%)
Minocycline	144	76 (52.8%)	68 (47.2%)
Rifampicin	144	99 (68.8%)	45 (31.2%)
Novobiocin	144	143 (99.3%)	1 (0.7%)
Colistin	144	140 (97.2%)	4 (2.8%)
Nalidixic acid	144	144 (100%)	0.0 (0%)
Tigecycline	144	143 (99.3%)	1 (0.7%)
Nitrofurantoin	144	143 (99.3%)	1 (0.7%)
Teicoplanin	144	141 (97.9%)	3 (2.1%)

## Discussion

Antibiotic resistance remains a major cause of mortality in bacterial infections across the age spectrum. Septicemia remains a major problem in developing countries including Pakistan. Irrational use of antibiotics has led to high levels of resistance among bacteria responsible for septicemia. Empiric antibacterial therapy should follow from most likely organisms and their known local resistance patterns. The present study provides data on causative organisms and their susceptibilities to commonly used antibacterials. The present study indicates that MRSA was the most common causative organism (37.5% of sepsis cases). Skin contaminants were found in 17.6%, and MRSA accounted for 14.8% cases. The result of the present study is consistent with other studies that found *Staphylococcus aureus* to be the most commonly identified pathogen [15-16]. Studies on adult septic patients indicate that *Klebsiella pneumonia* (8.14%) and *Escherichia coli *(4.65%) were the most frequently detected bacteria [16]. In contrast, our study showed that *Klebsiella *was detected in 3.4% and *Escherichia coli *in 11.4%.

Resistance and sensitivity pattern reveals that the majority of bacteria were resistant to common antibiotics used in general practices. There was no single antibiotic that has >70% susceptibility. Results indicate that in the penicillin group, efficacies of piperacillin/tazobactam and ampicillin/sulbactam were 21.5% and 14.6%, respectively. This susceptibility proportion was lower than that reported by Pradipta et al. in Indonesia (66.7% and 60.0% efficacies for piperacillin/tazobactam and ampicillin/sulbactam, respectively) [17]. In the present study, among the cephalosporins, cefoxitin had the highest susceptibility (28.5%).

Among fluoroquinolones, only 41.0% of samples were susceptible to ciprofloxacin and 24.5% to levofloxacin. The results of the present study were concordant with findings by Pradipta et al [17]. The present study reports global values for sensitivities by antibacterials rather than for each identified organism. Based on published studies, ciprofloxacin resistance is common in *Staphylococcus aureus* and *Enterococci* (50% and 33.3%, respectively). Among gram-negative microorganisms, ciprofloxacin resistance in *Acinetobactericia*, *Salmonella typhi*, *Escherichia coli*, and *Pseudomonas aeruginosa* against ciprofloxacin is reported as 9.1%, 6.3%, 26.1%, and 20%, respectively [18].

Among aminoglycosides, amikacin and gentamycin were found to be effective in 56.3% and 47.2% of septic cases, respectively. There are controversial findings about the resistant pattern of bacteria causing septicemia. A study conducted on bacterial isolate indicates that 55.56% of gram-negative bacteria were resistant to amikacin [19]. In such condition, it is recommended that aminoglycoside could be given in high dose. A study from Bangladesh among neonates with septicemia indicates that both gentamicin and amikacin have sensitivity pattern against gram-negative and gram-positive bacteria, ranging from 90% to 100% [20]. Another study reported that 75.0% of gram-positive isolates in septic patients show resistance to gentamicin [21]. This indicates that the sensitivity pattern varies across the countries depending on the use of antibiotics, immunity, and status of health care system as a whole. Thus, a local antibiogram broadly disseminated to clinicians is needed for optimal empiric therapy.

## Conclusions

Antibacterial resistance among bacteria-causing sepsis was alarmingly widespread. No single agent demonstrated bacterial susceptibility greater than 60%, with the majority being much lower. Possible reasons for this may include excessive use prophylactic antibiotics along and easy availability of over-the-counter drugs without prescriptions. A review on the use and availability of antibiotics is therefore highly recommended.
